# Orthogonally regulated multi-timescale reservoir and linear nonvolatile readout in a vertical transistor for dynamic machine vision

**DOI:** 10.1038/s41377-026-02420-4

**Published:** 2026-11-09

**Authors:** Qinyong Dai, Hangyuan Cui, Yishan Cao, Kai Chen, Chao Ai, Qijing Wang, Shunwei Zhu, Wenhao Wang, Zebo Fang, Bo Yao, Mengjiao Pei, Yi Shi, Yun Li

**Affiliations:** 1 Zhejiang Engineering Research Center of MEMS, Shaoxing University, Shaoxing, China; 2 National Laboratory of Solid-State Microstructures, School of Electronic Science and Engineering, Collaborative Innovation Center of Advanced Microstructures, Nanjing University, Nanjing, China; 3 Department of Applied Physics, The Hong Kong Polytechnic University, Hong Kong, China

**Keywords:** Photonic devices, Imaging and sensing, Optoelectronic devices and components

## Abstract

Implementing analog reservoir computing (RC) at the edge is fundamentally constrained by conflicting hardware requirements for the reservoir (volatile, nonlinear) and readout (nonvolatile, linear) layers. Here, we resolve this dilemma by monolithically integrating a multi-timescale photoelectric reservoir and a linear ferroelectric readout within a single vertical organic field-effect transistor (Fe-vOFET). This architecture functionally decouples photocarrier dynamics from ferroelectric polarization, allowing orthogonal optimization of reservoir dynamics and synaptic weights. In the optical mode, the Rubrene/F_16_CuPc heterojunction underpins reservoir dynamics, exploiting wavelength-dependent relaxation kinetics to construct multi-timescale grouped reservoirs that project inputs into high-dimensional spatiotemporal states. Conversely, in the electrical mode, the metal–ferroelectric–metal–insulator–semiconductor (MFMIS) stack governs readout functionality by mitigating interface trapping, translating remnant polarization into linear, symmetric, nonvolatile weight updates (endurance >10^6^, retention >3000 s, asymmetry nonlinearity 0.31 ± 0.05). The vertical-channel geometry is pivotal, simultaneously amplifying optical feedback and boosting the on/off ratio to ~10^7^, which is essential for high-fidelity weight mapping. Leveraging this dual-mode operation, we demonstrate a compact RC system achieving >90% accuracy in dynamic human-action recognition. This work establishes a scalable device paradigm that unifies dynamical richness with programming precision, paving the way for energy-efficient, all-in-one dynamic machine vision systems.

**Figure:**
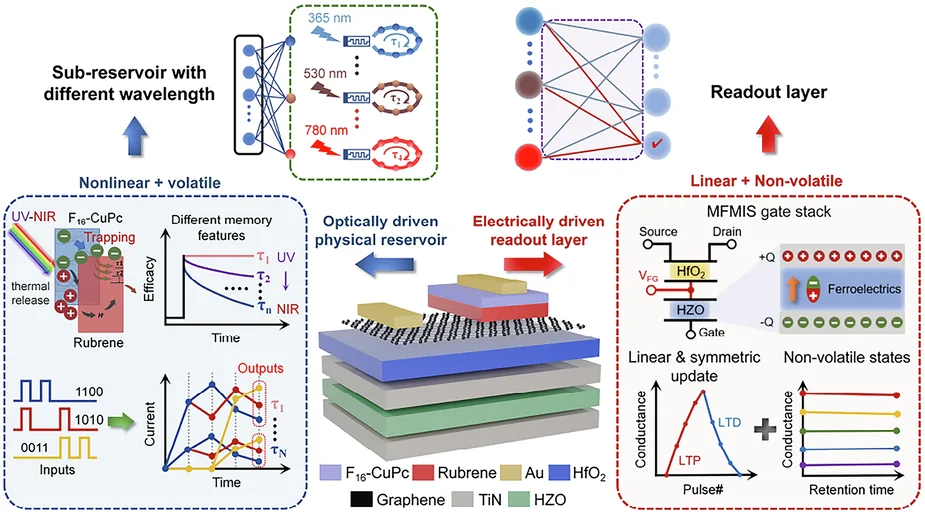

## Abstract (web summary)

A dual-mode Fe-vOFET integrates multi-timescale photoelectric reservoirs and linear nonvolatile ferroelectric readout, enabling compact, efficient dynamic machine vision with high recognition accuracy.

## Introduction

Artificial intelligence (AI) has revolutionized perception and cognition through deep neural networks (DNNs)^1–4^. Yet, for temporal signal processing in dynamic environments, conventional DNNs remain computationally prohibitive and energy-intensive, severely restricting their deployment in edge and autonomous systems^5,6^. Analog reservoir computing (RC) has emerged as a compelling alternative by keeping the recurrent connections of the reservoir fixed and training only a lightweight linear readout, thereby drastically reducing the learning cost^7–9^. Functionally, the reservoir projects sequential inputs into a high-dimensional state space through nonlinear transient dynamics, while the readout maps these states to target outputs via a linear combination, making RC particularly adept at spatiotemporal pattern recognition tasks such as dynamic machine vision.

Despite its algorithmic efficiency, the hardware implementation of RC faces a fundamental physical paradox: the reservoir and the readout layer impose conflicting requirements on device properties (Fig. S1, Supporting Information)^10,11^. The reservoir necessitates nonlinear input-to-state mapping and volatile dynamics with finite relaxation times to capture temporal dependencies (fading memory)^12,13^. In stark contrast, the readout demands linear, symmetric, and nonvolatile conductance modulation to ensure precise weight programming and stable inference^14,15^. Current implementations often resort to spatially separating these functions into distinct materials or device components, which inevitably compromises integration density and system compactness^16–18^. While single-device multifunctional approaches have been explored, they typically suffer from a performance trade-off: optimizing for volatile dynamics often degrades the nonvolatile retention or linearity required for the readout, and vice versa^19–21^. Specifically, on the reservoir side, limited carrier-relaxation dynamics restrict the diversity of accessible states, leading to overlapping temporal responses to complex input sequences^6,19–22^. On the other hand, nonideal conductance characteristics, including limited linearity, asymmetry, and insufficient retention and endurance, degrade weight precision and stability^23,24^. This often necessitates auxiliary write and verify circuitry, increasing system complexity and power consumption. Consequently, a unified architecture capable of orthogonally optimizing dynamical richness and programming precision within a single physical footprint remains a critical missing link for scalable neuromorphic hardware.

Recent progress in ferroelectric neuromorphic devices has highlighted their promise for artificial synapses, in-memory computing, and integrated sensory hardware, owing to their intrinsic nonvolatility, low-energy programming, and analog programmability^1,6,14,19,25^. These features are especially attractive for the readout layer, where stable and precise weight storage is required. Nevertheless, current ferroelectric device platforms still face important limitations, including nonideal update linearity and symmetry, device variation, and the difficulty of unifying stable memory with rich transient dynamics in a single-device architecture^5^. Thus, the key challenge is not simply to incorporate ferroelectricity into neuromorphic hardware, but to use it in a way that complements, rather than compromises, the volatile nonlinear dynamics needed for RC. This consideration motivates our strategy of coupling a linear nonvolatile ferroelectric readout with a nonlinear volatile optical reservoir through physically distinct control mechanisms.

To bridge this gap, we report a ferroelectric vertical organic field-effect transistor (Fe-vOFET) that functionally unifies a multi-timescale photoelectric reservoir and a linear ferroelectric readout. Our central design strategy lies in the physical orthogonalization of control mechanisms. Specifically, we exploit photocarrier trapping kinetics to define volatile reservoir states, while independently utilizing ferroelectric polarization switching to program nonvolatile readout weights. This structural decoupling ensures that the reservoir dynamics and memory retention can be optimized separately without interference (Fig. S2, Supporting Information). In operation, the optical mode leverages a broadband Rubrene/F_16_CuPc heterojunction to generate wavelength-dependent relaxation transients, thereby enabling grouped RC that significantly enriches the temporal feature space. Conversely, the electrical mode employs a robust metal–ferroelectric–metal–insulator–semiconductor (MFMIS) gate stack to mitigate interface trapping, thereby enabling the faithful transduction of ferroelectric polarization switching into highly linear and symmetric conductance updates (*A*_pot_ = 0.80 ± 0.04, *A*_dep_ = 1.15 ± 0.08), accompanied by significantly enhanced endurance and retention (>10^6^ cycles, >3000 s). Furthermore, the vertical-channel architecture is instrumental to this performance, as it not only enhances optical feedback through strong field confinement but also provides the high conductance on/off ratio (∼10^7^) necessary for high-fidelity weight mapping. By implementing this unified architecture, we realize a compact dynamic machine vision system capable of recognizing complex human actions with >90% accuracy, underscoring the immense potential of the Fe-vOFET architecture as a robust hardware platform for energy-efficient dynamic machine vision.

## Results

**Fig. 1:**
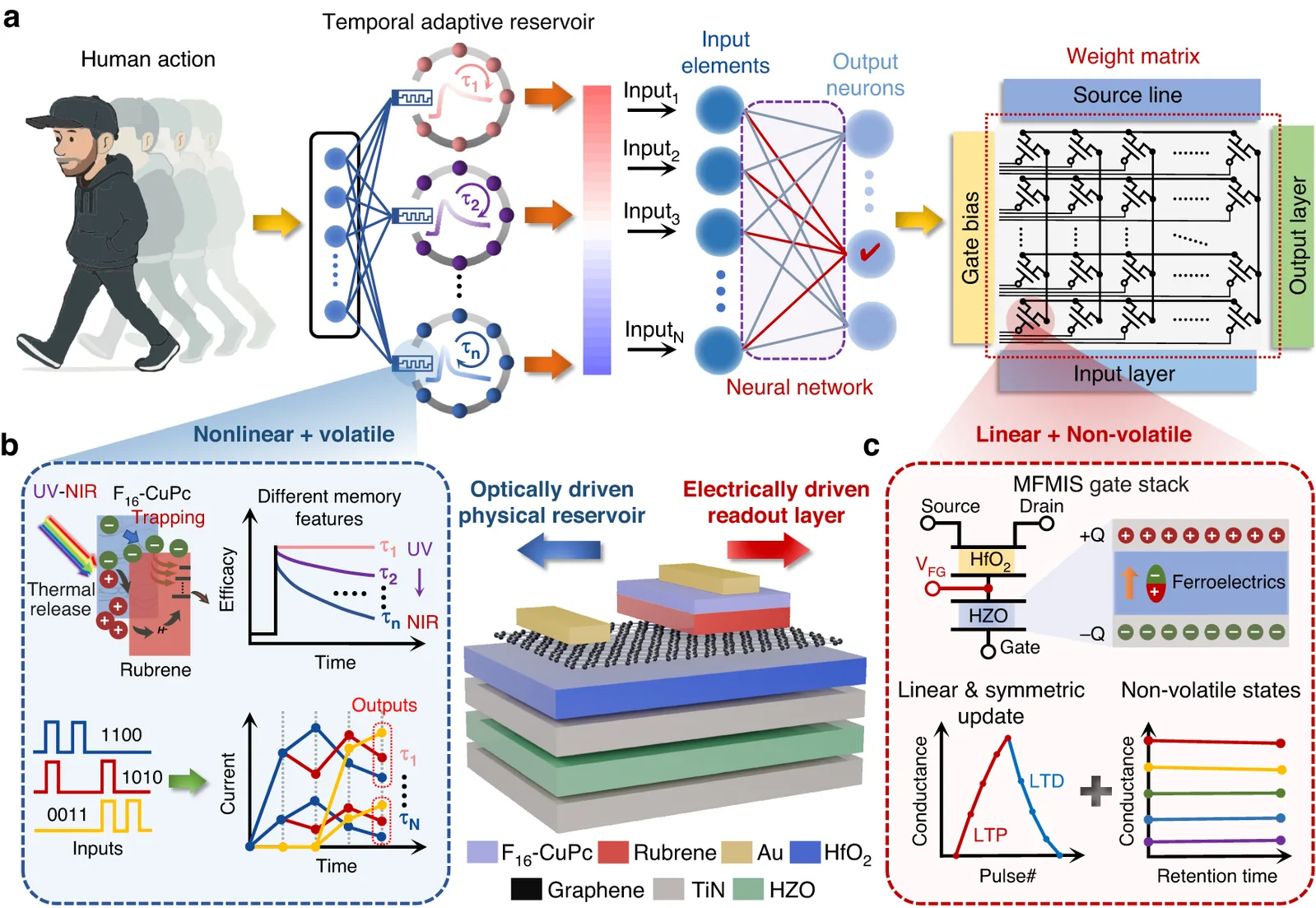
Reconfigurable photoelectric dual-mode Fe-vOFET for neuromorphic computing. **a** Schematic of an Fe-vOFET–based photoelectric RC system for human-action recognition: a front-end reservoir for nonlinear temporal mapping and a back-end ANN readout for classification, both realized with the same device in distinct modes. **b** Optically driven operation: broadband excitation yields wavelength-dependent relaxation, enriching reservoir states for multi-wavelength grouped RC. **c** Electrically driven operation: reversible ferroelectric polarization enables linear, symmetric, nonvolatile conductance updates suitable for precise weight programming and stable inference

To physically instantiate the proposed orthogonal optimization strategy for dynamic machine vision (Fig. 1a), we developed a Fe-vOFET that monolithically integrates a vertical-channel architecture with a robust MFMIS gate stack. The device fabrication and characterization details are provided in the Experimental Section, Figs. S3–S5, and Note S1 (Supporting Information). This architecture supports two complementary operational modes: an optical reservoir mode governed by the photocarrier dynamics of a Rubrene/F_16_CuPc heterojunction (Fig. 1b), and an electrical-readout mode programmed via ferroelectric switching in the HZO-based MFMIS stack (Fig. 1c).

**Fig. 2:**
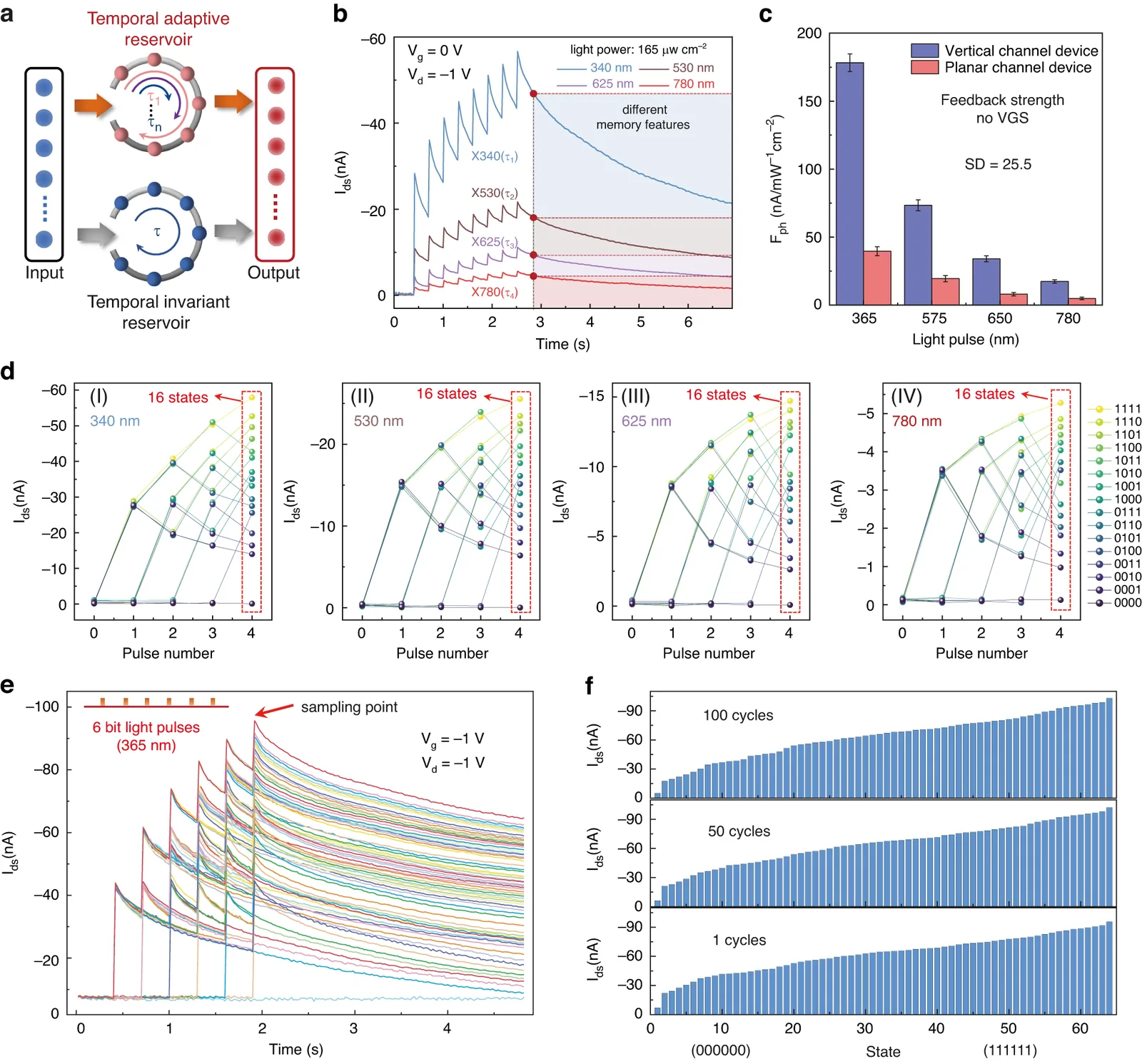
Optical operation of the Fe-vOFET for reservoir functionality. **a** Conceptual illustration of a multi-timescale reservoir, where the recursive RC structure captures temporal correlations in sequential inputs. **b** Wavelength-dependent optical mapping of physical node responses. **c** Comparison of feedback strength between planar and vertical geometries. **d** Transient *I*_d_ responses under sixteen representative multi-wavelength optical pulse sequences, showing effective expansion of the reservoir-statespace. **e** Transient current responses to six-bit optical pulse trains (six-bit reservoir-state encoding). **f** Statistical distributions of the sampled current values at the end of the sixth pulse, showing clear separability of the reservoir states

In conventional single-timescale RC systems, physical nodes typically rely on uniform carrier-relaxation dynamics to project sequential inputs, fundamentally restricting the accessible spatiotemporal feature space^16,25–27^. While external masking is widely employed to emulate node differentiation by redistributing input signals, this extrinsic modulation does not alter the intrinsic kinetics of the device. Consequently, the physical relaxation time constant (*τ*) remains invariant, which limits the diversity of intrinsic temporal dynamics and imposes additional circuit complexity. Effective grouped RC, by contrast, necessitates physical nodes with intrinsically diverse relaxation times and strong feedback coupling to enable parallel multi-timescale processing (Fig. 2a). Guided by this principle, we integrated a broadband-absorbing Rubrene/F_16_CuPc heterojunction into a vertical-channel architecture to extend the relaxation time distribution and physically enhance feedback coupling.

To evaluate the device as a physical reservoir node, we subjected the Fe-vOFET to optical pulse trains across the UV-NIR spectrum. The resulting transient currents exhibit nonlinear conductance evolution with volatile dynamics, fulfilling the critical requirement for time-dependent representation (Fig. 2b). Crucially, the relaxation kinetics are intrinsically governed by the incident photon energy: stimuli at different wavelengths trigger distinct decay profiles, yielding wavelength-selective transient dynamics. This spectral diversity allows a single device to project multi-wavelength optical sequences into orthogonal reservoir subspaces (e.g., X340τ_1_–X780τ_4_), thereby enabling multi-wavelength grouped RC. This strategy offers a hardware-efficient pathway to broaden the temporal feature space and enhance the computational dimensionality of the reservoir. Mechanistically, these optically driven transients originate from the interplay of photocarrier trapping and release within the Rubrene/F_16_CuPc heterojunction channel^28^. Under illumination, photogenerated electrons are captured by interfacial traps and localized states, while holes accumulate *via* the photogating effect, inducing a transient surge in *I*_ds_. Upon light removal, the gradual recombination of trapped electrons with channel holes dictates the slow decay of *I*_ds_, creating the short-term memory that underpins the volatile reservoir dynamics (Fig. S6, Supporting Information). Since the trapping and detrapping kinetics are energy-dependent, different wavelengths naturally drive distinct relaxation time constants, effectively encoding temporal information into the decay rate. Beyond material kinetics, the vertical-channel architecture (channel length ≈ 255 nm set by the active-layer thickness) plays a pivotal role in amplifying these dynamics. Compared to planar counterparts, the vertical geometry imposes a potent, non-uniform electric field that accelerates photocarrier separation and suppresses non-radiative recombination (Fig. S7, Supporting Information)^29^. This geometric confinement significantly enhances the feedback strength and broadens the spatial response profile (Fig. 2c). Furthermore, the monolayer graphene bottom electrode minimizes gate-field screening to ensure efficient optical modulation, while the substantial Schottky barrier at the graphene/Rubrene interface suppresses off-state leakage, yielding robust switching and electrical stability under repeated optical programming (Note S2 and Fig. S8, Supporting Information). The effective operating timescale of the optical reservoir in the present work is in the tens-of-milliseconds regime, as the optical pulse width used here is 50 ms. This timescale is mainly determined by the interplay of photocarrier trapping and relaxation kinetics, which govern the fading-memory behavior required for RC. In principle, the short carrier transport path in the vertical channel is favorable for faster operation and could enable shorter optical pulse intervals. However, reducing the pulse interval also lowers the photocurrent amplitude, thereby weakening the contrast and richness of the reservoir states. The effective operating speed of the optical reservoir is therefore set by a balance between transport efficiency and the need to preserve sufficiently distinguishable transient states for temporal information processing.

To further quantify the short-term memory behavior of the optical reservoir, we performed EPSC and PPF measurements at 340, 530, 625, and 780 nm (Fig. S9, Supporting Information). The device exhibits a clear EPSC response under a single optical pulse, followed by gradual decay after stimulus removal, and a pronounced PPF effect under paired optical pulses, indicating the existence of a residual internal state. Double-exponential fitting of the EPSC decay curves yields characteristic time constants of *τ*_1_ = 0.347, 0.308, 0.239, and 0.182 s and *τ*_2_ = 3.61, 2.23, 1.93, and 1.51 s for 340, 530, 625, and 780 nm, respectively. These results, together with the wavelength-dependent PPF behavior, further confirm that the device possesses multi-timescale fading-memory dynamics suitable for temporal information processing in the photonic reservoir. In addition, the device still maintains clear light-triggered transient responses even at an ultralow drain bias of −25 μV, highlighting its potential for ultralow-power optical reservoir operation (Fig. S10, Supporting Information).

We systematically analyzed the dynamic evolution of channel conductance under optical input sequences to validate the reservoir’s temporal processing capacity. As depicted in Fig. 2 d, the Fe-vOFET generates distinct transient trajectories for all 16 permutations of a 4-bit pulse train (165 μW cm^−2^, 20 ms), confirming precise sequence discrimination. Functionally, multi-wavelength modulation serves as a dimensionality expander, broadening the overall state space by creating orthogonal response clusters without compromising the intrinsic separability within each wavelength group (Fig. S12, Supporting Information). The device’s high-resolution temporal encoding was further substantiated using a 6-bit programming sequence (from ‘000000’ to ‘111111’). The Fe-vOFET successfully resolved 64 distinct conductance levels, demonstrating its ability to map complex optical inputs into a broad spectrum of distinguishable transient memory states (Fig. 2e). For the six-bit optical input sequences, the relevant criterion is the distinguishability of the transient reservoir states at the predefined sampling point used for readout, rather than complete separation of the full transient trajectories at every moment. To examine this point quantitatively, we performed repeated measurements of the six-bit optical pulse responses and extracted the current values at the end of the sixth pulse (Fig. 2 f). The statistical results show that the reservoir states associated with different six-bit input sequences are generally distinguishable at this readout point, supporting the proof-of-concept implementation of six-bit transient-state encoding. At the same time, some neighboring states remain relatively close, suggesting that further enhancement of the photo response, for example, through interface engineering, would be beneficial for increasing the state margin. Beyond spectral tuning, the gate voltage (*V*_g_) provides an additional electrical degree of freedom to optimize state separation. Fig. S13 illustrates representative “1011” sequences under coupled *V*_g_ and wavelength modulation. By modulating the Schottky barrier height at the active-layer/source interface, *V*_g_ effectively tunes the carrier injection efficiency and channel conductance baseline. This synergistic control yields memory currents with diversified amplitudes and relaxation offsets, thereby enriching the reservoir dynamics. Collectively, these results demonstrate that the Fe-vOFET creates a highly separable, high-dimensional reservoir space through coupled optical and electrical regulation, establishing a robust physical foundation for the subsequent pattern recognition tasks.

**Fig. 3:**
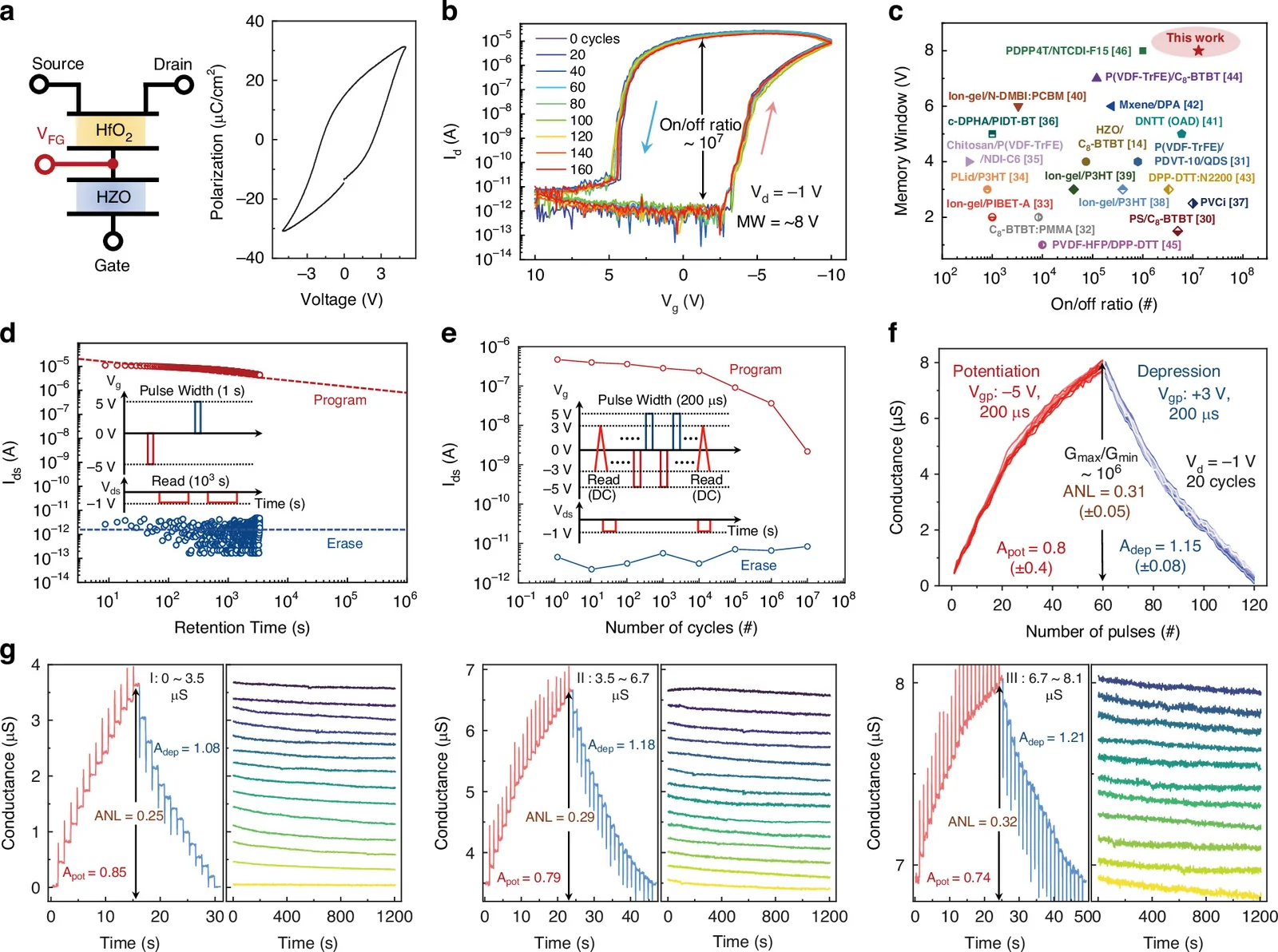
Electrical operation of the Fe-vOFET for readout functionality. **a** Simplified schematic of the MFMIS structure and typical *P*–*V* hysteresis loops of the TiN/HZO/TiN/HfO_2_/Rubrene/F_16_CuPc/Au capacitor obtained from PUND measurements. **b** Transfer characteristics during 100 consecutive cycles. **c** Benchmark comparison of on/off ratio and memory window with previously reported organic nonvolatile memories. **d** Retention characteristics of ON/OFF states. **e** Endurance performance under ±5 V, 200 μs program/erase pulses. **f** Cycle-to-cycle variation of 20 consecutive LTP/LTD operations. **g** LTP/LTD characteristics at different conductance ranges

Complementing the volatile optical reservoir, the readout layer necessitates linear, symmetric, and nonvolatile conductance modulation. To physically realize these rigorous requirements within the same device footprint, we integrated a robust MFMIS gate stack into the vertical transistor architecture (Fig. 3a). This configuration is specifically engineered to enhance electrostatic coupling while buffering the organic channel from the deleterious interface effects common in conventional ferroelectric/semiconductor junctions. Electrical characterization confirms the efficacy of this design strategy. As shown in Fig. 3b, the Fe-vOFET exhibits a clear counterclockwise hysteresis with a memory window of ~8 V, consistent with robust ferroelectric polarization reversal in the HZO layer. The hysteresis loop is highly symmetric around 0 V, implying balanced polarization switching with negligible contribution from interfacial charge trapping, which is a direct benefit of the MFMIS buffering effect. Notably, the device achieves a high on/off ratio of 1.8 × 10^7^ at *V*_d_ = −1 V. This exceptional switching performance is attributed to the vertical-channel geometry, which provides an ultrashort transport path and strong vertical-field confinement, effectively widening the conductance window for high-fidelity weight mapping. Figure 3c benchmarks our device against recent organic nonvolatile memories, highlighting its superior trade-off between memory window and switching ratio^14,30–46^. Reliability measurements further underscore the robustness of the Fe-vOFET. Retention tests (Fig. 3d) demonstrate that the ON and OFF states remain distinct and stable for over 3000 s, validating the nonvolatile capability required for long-term synaptic weight storage. In endurance assessments (Fig. 3e), the device sustains a robust memory window (>10^5^) after 10^6^ program/erase cycles (±5 V, 200 μs), indicating durability fully compatible with intensive neuromorphic learning operations. The former reflects the stability of the stored conductance states under the standard read condition, whereas the latter probes the robustness of ferroelectric switching under cyclic electrical stress. Based on the present data, the degradation after extended cycling is most likely associated with a combination of ferroelectric fatigue, interfacial charge trapping/detrapping, and accumulated electrical stress in the gate stack, which may gradually reduce the conductance window and make adjacent programmed states less distinguishable. Moving beyond binary storage, the Fe-vOFET demonstrates analog conductance modulation essential for synaptic emulation. Figure 3f depicts the conductance evolution under 20 consecutive cycles of potentiation and depression pulses. The conductance values were extracted 1 s after the end of each gate-voltage pulse, following programming pulses of −5 V for 200 μs and erasing pulses of +3 V for 200 μs. This readout delay was chosen to avoid the immediate transient disturbance after the programming/erasing event and to provide a more stable evaluation of the nonvolatile conductance state. The updates are highly reproducible, featuring bidirectional symmetry and minimal cycle-to-cycle fluctuation. Quantitative analysis confirms the improved linearity and symmetry, with extracted parameters of *A*_pot_ = 0.80 ± 0.04 and *A*_dep_ = 1.15 ± 0.08, along with a low asymmetry nonlinearity (ANL) of 0.31 ± 0.05 (details in Note S3, Supporting Information). Moreover, the device maintains consistent linear update characteristics across multiple conductance ranges, with each state exhibiting stability for over 1200 s without noticeable state-drift (Fig. 3g). To further quantify the reliability of the nonvolatile readout states, we also performed repeated-read statistics for each programmed conductance level (500 read points per state), which show well-separated conductance distributions with limited fluctuation (Fig. S15, Supporting Information). Although the present programming voltages (−5 V and +3 V) are higher than those typically used in scaled CMOS logic, they are associated mainly with state programming rather than routine logic-level signal transmission. In a practical hybrid system, such programming can in principle be handled by dedicated peripheral drivers or level-shifting circuits, while the readout operation itself can be performed at much lower bias. Further reduction of the programming voltage will be an important direction for future optimization of the ferroelectric stack and device geometry. These attributes confirm that the MFMIS stack successfully transduces polarization dynamics into precise, energy-efficient weight updates, eliminating the need for complex iterative write-verify circuitry.

The superior robustness and programming precision of the Fe-vOFET are structurally underpinned by the MFMIS gate stack. In conventional metal–ferroelectric–semiconductor architectures, direct contact between the ferroelectric layer and the semiconductor inevitably induces depolarization fields and interfacial charge trapping, leading to incomplete polarization switching and accelerated retention degradation (Fig. S16, Supporting Information). By contrast, the MFMIS configuration introduces an intermediate floating gate and a buffering dielectric to decouple these layers. This architecture enhances electrostatic coupling and provides sufficient induced charge to fully compensate for the ferroelectric polarization, while effectively isolating the active channel from interface traps. Such structural engineering ensures complete, stable polarization reversal and suppresses leakage currents, thereby securing the operational uniformity required for precise weight updates under our bias conditions. To assess the scalability of this architecture, we evaluated the device-to-device variability across 21 independently fabricated Fe-vOFETs (Fig. S18, Supporting Information). The statistical distribution reveals highly stable memory windows with a median on/off ratio of ~10^6^ and a variation coefficient below 15%. This observed variation is primarily ascribed to minor thickness fluctuations in the rubrene single crystals synthesized via physical vapor transport (PVT) and transferred graphene layers, suggesting that standardizing the transfer process and crystal selection could further suppress variability for large-scale integration. Collectively, these results establish the Fe-vOFET as a robust readout primitive. Its capability for accurate, nonvolatile, and linear weight programming perfectly complements the nonlinear, multi-timescale dynamics of the optical reservoir, thereby fulfilling the hardware prerequisites for the unified, compact RC system implemented for dynamic machine vision tasks.

The apparent difference between the volatile behavior in Fig. 2e and the improved nonvolatile behavior in Fig. 3g is expected, because the two figures correspond to different operating modes governed by different physical mechanisms. In the optical reservoir mode, the conductance response is dominated by photocarrier generation, trapping, and relaxation in the heterojunction channel, and therefore naturally relaxes back toward the baseline after the optical stimulus is removed. By contrast, in the electrical-readout mode, the conductance state is stabilized by remanent ferroelectric polarization in the MFMIS stack, which generates an internal electric field that continues to modulate the channel even after the external programming bias is removed. To further verify that the volatile reservoir function and the nonvolatile readout function can coexist in the same device platform without destructive interference, we examined the stability of programmed ferroelectric states under repeated optical stimulation (Fig. S19a, b, Supporting Information). After the device was first programmed into different conductance states by electrical pulses, repeated optical pulse stimulation was applied. Although transient photocurrent responses were superimposed during illumination, the conductance returned to the original programmed baseline after the optical pulses were removed, indicating that optical excitation does not erase or rewrite the programmed ferroelectric state, but only introduces a temporary photocarrier-induced modulation. This conclusion is further supported by PFM measurements, which show that electrical stimulation switches the ferroelectric domains, whereas light illumination does not induce an observable change in the ferroelectric phase contrast (Fig. S19c, Supporting Information). These results provide direct evidence that the reservoir and readout functions originate from physically distinct mechanisms and can therefore be optimized independently within the same Fe-vOFET platform. At the system level, the present architecture operates in mode-separated reservoir and readout configurations, rather than relying on simultaneous multifunctional operation in a single node. Reservoir and readout can thus be implemented in different arrays or submodules based on the same device technology, which is advantageous for compact integration and independent optimization of the two functions.

**Fig. 4:**
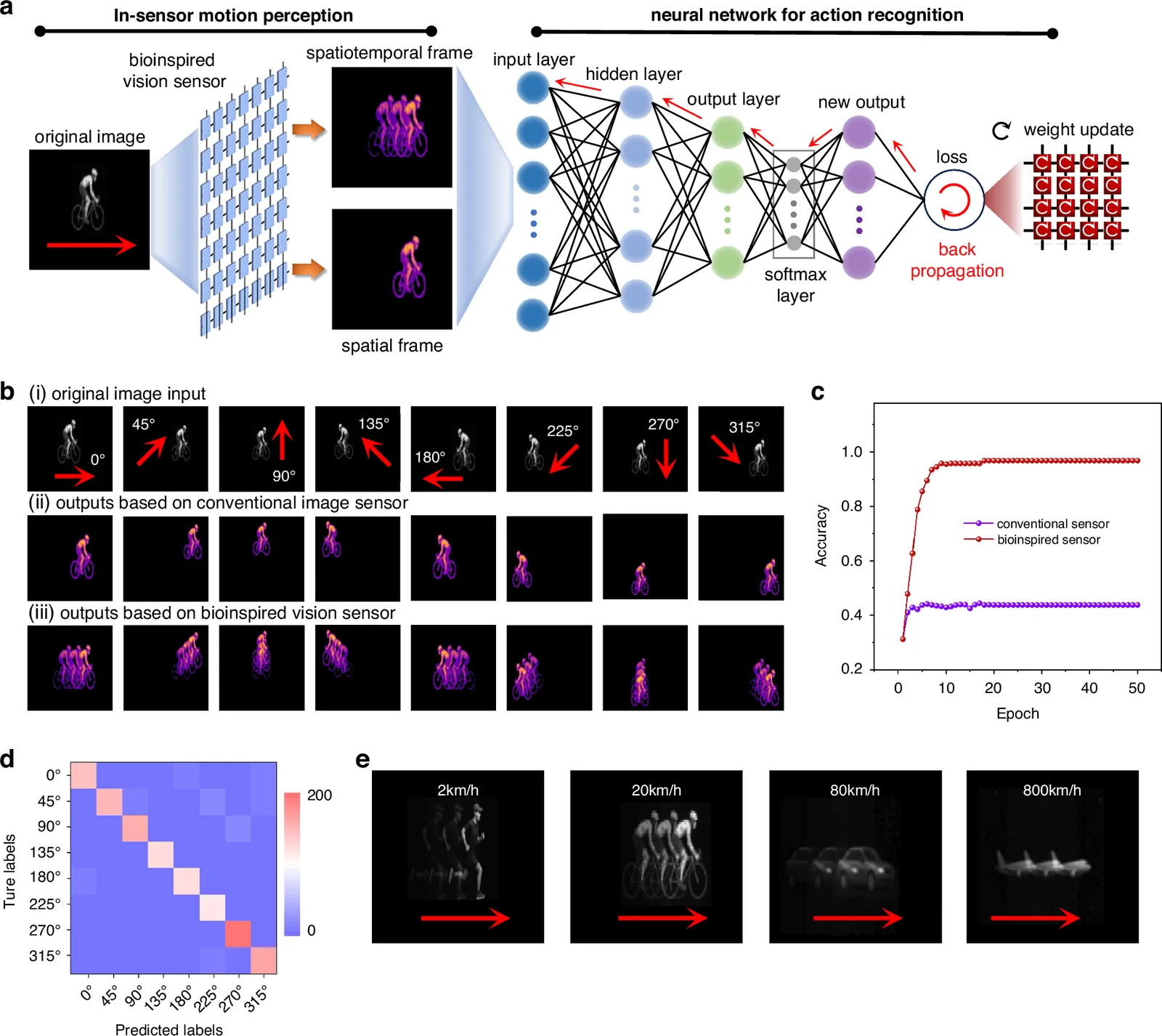
Fe-vOFET-based bio-inspired vision system for motion recognition. **a** Compact neural-network framework using either the bio-inspired sensor or a conventional image sensor. **b** Bicycle motion viewed from eight azimuth angles (i), and the corresponding outputs from the bio-inspired sensor (ii) and the conventional sensor (iii). **c** Recognition accuracy of the bio-inspired and conventional sensors during training. **d** Confusion matrix for trajectory recognition across the eight viewing angles. **e** Gate-tunable temporal encoding of motions with different characteristic speeds

Accurate and agile motion recognition is central to machine vision applications such as autonomous driving and intelligent surveillance. To further demonstrate the practical relevance of the proposed Fe-vOFET, we implemented a traffic-trajectory recognition task with the device operated in the optical mode. A custom video dataset was constructed, comprising eight motion categories corresponding to eight representative travel directions. Based on this dataset, we built a vision system in which a Fe-vOFET-based sensor array serves as the front-end encoder, while the encoded outputs are classified by a compact artificial neural network (Fig. 4a). As a reference, we also considered a baseline configuration using conventional image frames that contain only instantaneous spatial information without temporal accumulation, thereby mimicking the operation of a standard image sensor. Representative output frames from the Fe-vOFET-based bio-inspired vision sensor are shown in Fig. 4bii. Unlike the baseline frames, which resemble those produced by a conventional image sensor and record only the current stimulus at each acquisition time (Fig. 4biii), the Fe-vOFET-generated frames exhibit temporally compressed states that integrate the optical responses over the full motion sequence. As a result, the motion contour is directly preserved in a single compressed frame, enabling simultaneous encoding of spatial structure and temporal evolution. This spatiotemporal fusion at the sensing stage leads to a substantially more discriminative representation of motion patterns. The resulting advantage is clearly reflected in the recognition performance. As shown in Fig. 4c, d, the Fe-vOFET-based bio-inspired vision sensor converges within ~10 training epochs and achieves a final recognition accuracy of 97.2%, whereas the baseline saturates at only ~40% under the same network architecture and training protocol. These results highlight the importance of front-end temporal accumulation for efficient motion understanding.

In realistic traffic scenarios, target velocities can span a wide range, from slowly moving pedestrians to high-speed vehicles and aircraft. Accordingly, different sampling rates and temporal integration windows are desirable to balance recognition accuracy against computational cost. In the Fe-vOFET-based bio-inspired vision sensor, the effective temporal integration can be tuned by the gate bias *V*_g_, which modulates the photocarrier relaxation time. Using this gate-controlled tunability, motions at different speeds can be encoded into spatiotemporal frames acquired under different *V*_g_ conditions (Fig. S20, Supporting Information). As summarized in Fig. 4e, negative gate biases (for example, *V*_g_ = −3 V) prolong the integration window and are well suited for recognizing slow trajectories such as pedestrian or running motion, whereas positive gate biases (for example, *V*_g_ = +1.5 V) shorten the integration time and are more suitable for fast trajectories such as aircraft motion. This gate-programmable temporal summation at the sensory front-end enables the Fe-vOFET-based vision system to adapt to a broad range of real-world motion speeds while maintaining high recognition accuracy and allowing flexible trade-offs between sensing bandwidth and computational load.

**Fig. 5:**
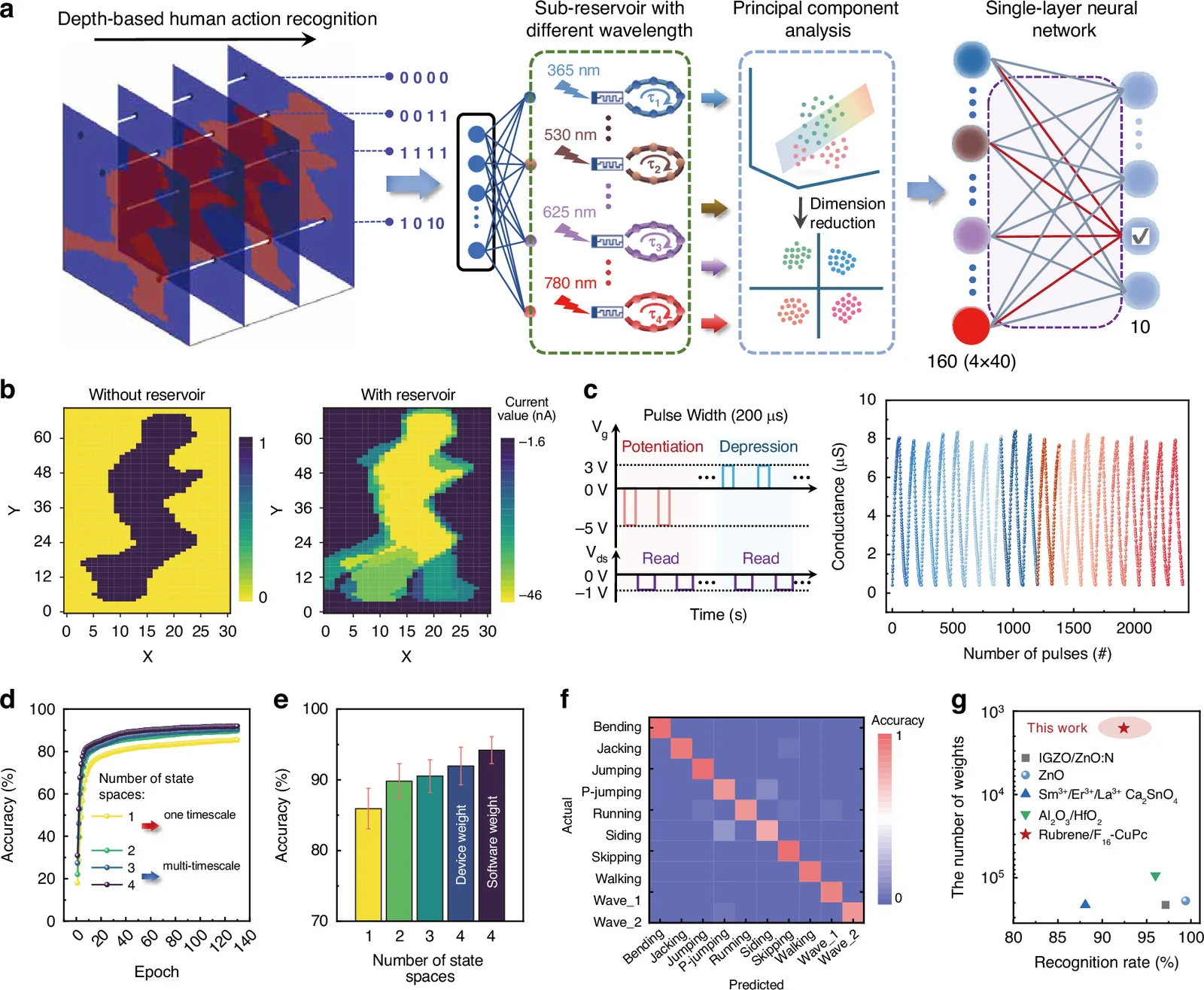
Human action-recognition based on Weizmann dataset using the Fe-vOFET-based photoelectric RC system. **a** Schematic of the RC framework integrating a multi-wavelength optical reservoir and linear electrical readout. **b** Comparison of photocurrent values at 340 nm with reservoir processing and without reservoir processing, corresponding to four schematic frames of the “run” action. **c** Conductance evolution showing linear and symmetric LTP/LTD behavior. **d**, **e** Effect of grouped sub-reservoir number on recognition accuracy. **f** Confusion matrix for the four-group configuration. **g** Comparison with reported RC systems based on the same dataset

To validate the efficacy of the proposed device-level strategy, we constructed a compact RC framework for dynamic machine vision, utilizing the multi-wavelength optical and electrical modes of the Fe-vOFET as the reservoir and readout, respectively (Fig. 5a). The system was benchmarked on the Weizmann human-action dataset, a standard task for assessing spatiotemporal pattern recognition. Original video clips were binarized and aligned, and four frames were uniformly sampled from each 13-frame sequence to capture a complete motion cycle, yielding 396 samples. Each frame was cropped to 70 × 30 pixels, providing standardized spatial inputs for reservoir processing. In this framework, video sequences of human actions are encoded as optical pulse trains and distributed to the Fe-vOFET *via* four parallel spectral paths. Leveraging the wavelength-dependent relaxation kinetics, the system processes the input stream concurrently through these distinct wavelength channels (340, 530, 625, and 780 nm), thereby creating four “sub-reservoirs”. Crucially, the output of each virtual node was derived directly from the experimentally measured optical conductance responses (Fig. 2 d) to ensure physical fidelity. To illustrate this dynamic mapping, Fig. 5b displays the evolving reservoir currents at 340 nm corresponding to the four-frame sequence of the ‘run’ action shown in Fig. 5a. This reservoir-enabled operation effectively accumulates temporal information over the motion cycle, contrasting sharply with the reservoir-free baseline, which only preserves the instantaneous state of the final frame, thereby failing to capture temporal dependencies. Because the resulting state matrices are high-dimensional and partly redundant, principal component analysis (PCA) was performed for each sub-reservoir to extract 40 principal components that capture the dominant spatiotemporal variation (Fig. S21, Supporting Information). These features from the four sub-reservoirs were concatenated into a 160-dimensional vector (4 × 40) and input to a single-layer artificial neural network (ANN) readout employing softmax for classification. Readout weights were optimized with Adam and mapped onto the experimentally extracted long-term potentiation (LTP) and long-term depression (LTD) characteristics of the Fe-vOFET after each training epoch, emulating the physical weight memory and iterative learning (Fig. 5c). Based on the readout architecture used in this task, an order-of-magnitude estimate of the readout-side electrical energy can also be obtained. For the 160 × 10 output layer adopted here, using a read-bias magnitude of 1 V, a representative current level of 50 nA, and a weight-update pulse width of 200 μs, the energy is estimated to be on the order of 10 nJ per inference for the output layer, corresponding to ~10 pJ per cell operation. This estimate is intended to provide a task-level device-based order of magnitude and does not include peripheral circuitry, driver overhead, or losses associated with the external optical source. For statistical rigor, the system underwent repeated random sub-sampling validation (50 iterations), where 70% of the samples were randomly assigned for training and 30% for testing in each round, with the mean accuracy reported as the final result. The evolution of the classification accuracy with training epoch is shown in Fig. S22a, while the corresponding validation loss is presented in Fig. S22b (Supporting Information). The accuracy increases rapidly at the early stage of training and then gradually approaches a stable regime at longer epochs. Meanwhile, the validation loss decreases continuously and exhibits a clear trend toward convergence, indicating that the training remains stable without obvious late-stage divergence. These results provide a more complete view of the optimization behavior of the readout network. Because different optical wavelengths naturally induce distinct photocarrier relaxation kinetics, the number of sub-reservoir groups also determines how many temporal scales are involved in the encoding process. Classification results further underscore the critical role of multi-timescale reservoir dynamics. The reservoir-free baseline, lacking temporal accumulation capability, achieves a recognition rate of only 67.8% (Fig. S23, Supporting Information). More importantly, restricting the system to a single sub-reservoir (one group) is physically equivalent to operating under a restricted single-timescale condition, because only one wavelength-dependent relaxation channel is retained. Under this condition, the reservoir can capture only limited temporal diversity, resulting in a lower recognition accuracy of 85.46%. As additional sub-reservoir groups are introduced, corresponding to the incorporation of progressively richer wavelength-dependent temporal scales, the classification accuracy increases accordingly and reaches 91.95% in the four-group case (Fig. 5d, e). This comparison indicates that the multi-timescale dynamics provide complementary temporal features that are beneficial for improving computational performance. Notably, the accuracy achieved using the physical LTP/LTD characteristics of the device is comparable to that of software-optimized weights, confirming that the high linearity and symmetry enabled by the MFMIS readout are sufficient to support precise learning. The confusion matrix for the four-group configuration (Fig. 5f) reveals accurate discrimination among 10 motion classes with minimal inter-class interference, further verifying the robustness of the system. Compared to state-of-the-art RC systems evaluated on the same dataset, our approach attains comparable accuracy while reducing weight-related computation by nearly two orders of magnitude, enabled by an integrated optoelectronic platform with a compact architecture and balanced performance across the reservoir and the readout (Fig. 5g)^47–50^. The generality of the proposed photoelectric RC framework was also validated on MNIST handwritten-digit recognition, a static image benchmark, where classification accuracy remained high (Fig. S24, Supporting Information). To further examine the robustness of the framework on more challenging static visual data, we additionally evaluated it on CIFAR-10 color-image recognition (Fig. S25, Supporting Information). The RGB channels of each image were first processed separately to extract HOG features, which were then combined into a one-dimensional feature sequence and encoded into reservoir inputs using the same 4-bit mapping scheme (0000–1111) as used above. After reservoir processing, PCA and ANN-based readout were applied for classification. Despite the increased complexity of color images and inter-class variability, the system maintains good recognition performance on four representative CIFAR-10 categories. This result further supports that the proposed photoelectric RC framework can be extended beyond simple grayscale benchmarks to more challenging visual recognition tasks. Together, these results demonstrate that the proposed photoelectric RC framework is applicable across both simple and more challenging visual recognition tasks.

Finally, to place the present Fe-vOFET more clearly in the context of recent neuromorphic vision and hardware RC studies, Table S1 compares representative device platforms in terms of integration level, reservoir dynamics, readout characteristics, scalability-related considerations, and demonstrated tasks. Existing approaches generally fall into two categories. In one category, the reservoir and readout are implemented by separate devices or material platforms, which can provide strong task performance but often at the cost of increased fabrication complexity, interconnect overhead, and reduced system compactness. In the other category, single-device multifunctional systems have been explored to improve integration density, but they often face a trade-off between the volatile nonlinear dynamics required for RC and the linear, symmetric, and nonvolatile conductance modulation required for accurate readout. In many such cases, the physical mechanisms supporting the two functions are not sufficiently separated, so improvements in one mode may compromise performance in the other. By contrast, the Fe-vOFET reported here adopts a monolithic but mode-separated architecture, in which the optical channel provides a nonlinear volatile reservoir with multi-timescale dynamics, while ferroelectric gating provides a linear nonvolatile readout with stable weight-update characteristics. This physical separation between photocarrier dynamics and ferroelectric polarization allows the two functions to be optimized independently within the same device platform. At the system level, this strategy is compatible with implementation in reservoir and readout arrays built from the same device technology, which is advantageous for compact integration and future scaling. In addition, the present framework has been validated not only on static image tasks, but also on dynamic vision and more challenging color-image recognition benchmarks, further highlighting its versatility across different computational scenarios.

## Discussion

In summary, we have validated a device-level strategy that successfully harmonizes the contradictory hardware demands of RC within a single Fe-vOFET architecture. By enabling the orthogonal regulation of photocarrier kinetics and ferroelectric polarization, our design achieves a unified dual-mode operation where volatile temporal feature extraction and nonvolatile weight storage coexist without mutual interference. Of particular note, the vertical-channel geometry serves as a physical cornerstone for this performance, serving the dual purpose of amplifying optical feedback loops for dynamical diversity and securing the high conductance on/off ratio (~10^7^) required for accurate weight mapping. The efficacy of this compact framework is evidenced by the >90% accuracy attained in complex human-action-recognition tasks (Weizmann), confirming that structural integration does not necessitate a trade-off in computational fidelity. Ultimately, this work provides a blueprint for balancing dynamical richness with readout precision in a scalable physical footprint. By resolving the fundamental conflict between volatile reservoir dynamics and memory retention, the Fe-vOFET paradigm paves the way for the next generation of adaptive, energy-efficient, and all-in-one dynamic machine vision systems.

## Materials and methods

### Fabrication of ferroelectric vertical-channel organic field-effect transistors

Fe-vOFETs were fabricated through a sequential multistep process. Si/SiO_2_ substrates were sequentially ultrasonicated in acetone, isopropanol, and deionized water (15 min each) and then nitrogen-dried. A 20 nm TiN bottom gate was deposited by electron-beam evaporation (EBE), followed by deposition of a 16 nm Hf_0.5_Zr_0.5_O_2_ (HZO) layer via atomic layer deposition (ALD) at 200 °C using TDMA–Hf and TDMA–Zr precursors with H₂O as the oxidant. A 20 nm TiN floating gate was subsequently deposited by EBE atop the HZO layer, and rapid thermal annealing was performed at 450 °C for 30 s under nitrogen to crystallize HZO, during which the TiN acted as a capping electrode to promote ferroelectric phase formation and suppress oxygen loss. A 10 nm HfO_2_ interfacial dielectric was then grown by ALD at 200 °C using TDMA–Hf and O_3_ as the oxygen source, completing the MFMIS heterostructure.

Monolayer graphene was mechanically exfoliated and transferred onto the HfO_2_ surface to serve as the shared bottom source electrode of the vertical channel. Au contact pads were transferred onto the graphene to provide low-resistance probe terminals and facilitate external electrical connection. High-quality rubrene single crystals were synthesized via PVT: rubrene powder (purity >99%, Aladdin Chemicals) was placed on a clean Si substrate in the sublimation zone (~340 °C) of a quartz-tube furnace under 20 sccm argon flow. The crystals condensed downstream at ~220 °C and were collected near the tube end by positioning the source 10.5–12.0 cm from the outlet to enable convenient transfer. The obtained rubrene crystals were then transferred onto the graphene/HfO_2_ stack using a flexible polymer micro-probe. Subsequently, a 10 nm copper hexadecafluorophthalocyanine (F_16_CuPc) film was thermally evaporated onto the rubrene surface to form a planar organic heterojunction. Finally, top electrodes were laminated onto the overlapping region between the rubrene/F_16_CuPc heterostructure and the graphene layer, completing the Fe-vOFET fabrication.

### Characterization and measurements

The electrical and photoelectrical characteristics of the Fe-vOFETs were systematically evaluated using a semiconductor parameter analyzer (Agilent B1500) integrated within a closed-loop low-temperature probe station under a vacuum pressure of ~10^−2^ Pa. All electrical measurements were performed at room temperature unless otherwise specified. Optical pulse stimulation was provided by fiber-coupled LEDs at representative wavelengths of 340, 530, 625, and 780 nm. The fiber-coupled configuration provides stable optical output, well-confined light delivery, and well-defined emission spectra for reliable wavelength-dependent transient photo response measurements. The corresponding emission spectra of the four LEDs are provided in Fig. S14. The irradiance of each wavelength was calibrated before measurement to ensure consistent optical power density across different illumination conditions.

The surface morphologies of rubrene single crystals, monolayer graphene, F_16_CuPc, HZO, and HfO_2_ thin films were characterized using an atomic force microscope (AFM, Park Systems) operated in tapping mode. The ferroelectric properties of the HZO layer were quantitatively analyzed using piezo response force microscopy (PFM, Oxford Instruments) and a ferroelectric test system (Oxford Electronics). The PFM amplitude and phase mappings confirmed robust ferroelectric switching, while polarization–electric field (*P*–*E*) hysteresis loops were recorded to assess remnant polarization, coercive field, and switching stability. These combined electrical, optical, and structural characterizations provided comprehensive insight into the coupling mechanisms and performance metrics of the Fe-vOFETs.

### Implementation of the RC tasks

For all application-level evaluations, the experimentally measured optical responses of the Fe-vOFET reservoir were used as the physical signals directly entering the computation framework. In the Weizmann action-recognition task, the transient optical conductance/current responses measured from the Fe-vOFET under the corresponding optical stimulation sequences were directly used as the reservoir states, and then assembled into high-dimensional state matrices. The subsequent PCA, feature concatenation, and single-layer ANN readout with Adam optimization were implemented in software. The optimized readout weights were then mapped onto the experimentally extracted LTP/LTD characteristics of the Fe-vOFET to emulate physical weight storage and iterative learning. For the other benchmarks, the same hybrid framework was adopted, while only the front-end input encoding was task-dependent. For MNIST, the binarized image pixels were converted into input pulse trains and sequentially applied to the Fe-vOFET-based physical reservoir. For CIFAR-10, HOG features were extracted separately from the RGB channels, combined into a one-dimensional feature sequence, binarized, and then encoded into reservoir inputs using the same 4-bit mapping scheme as used in the main text. In all cases, the resulting reservoir-encoded feature vectors were processed by software-based PCA and ANN readout unless otherwise specified.

## Supplementary information

Supplementary Information

## Data Availability

The data that support the findings of this study are available from the corresponding author upon reasonable request.
