# Supplementary material for: Orthogonally regulated multi-timescale reservoir and linear nonvolatile readout in a vertical transistor for dynamic machine vision
Source: Light Sci Appl. 2026 Nov 9;15:398. doi: 10.1038/s41377-026-02420-4 (PMC13745582; doi:10.1038/s41377-026-02420-4)
Supplement: Supplementary file 1 [file 41377_2026_2420_MOESM1_ESM.docx]

Supplementary Information

Orthogonally Regulated Multi-Timescale Reservoir and Linear Nonvolatile Readout in a Vertical Transistor for Dynamic Machine Vision

*Qinyong Dai^1^, Hangyuan Cui^2^, Yishan Cao^2^, Kai Chen^2^, Chao Ai^2^, Qijing Wang^2^, Shunwei Zhu^1^, Wenhao Wang^1^, Zebo Fang^1^, Bo Yao^1^*, Mengjiao Pei^3^*, Yi Shi^2^*, and Yun Li^2^**

Q. Dai, S. Zhu, W. Wang, Z. Fang, B. Yao

^1^Zhejiang Engineering Research Center of MEMS, Shaoxing University, Shaoxing, 312000, China.

*Email: yaob_usx@163.com

H. Cui, Y. Cao, K. Chen, C. Ai, Q. Wang, Y. Shi, Y. Li

^2^National Laboratory of Solid-State Microstructures, School of Electronic Science and Engineering, Collaborative Innovation Center of Advanced Microstructures, Nanjing University, Nanjing 210093, China.

*Email: yshi@nju.edu.cn; yli@nju.edu.cn

M. Pei

^3^Department of Applied Physics, The Hong Kong Polytechnic University, Hong Kong SAR, China

*Email: mengjiao.pei@polyu.edu.hk

**Fig. S1.** Different hardware requirements for reservoir and readout layers.

**Fig. S2.** The physically decoupled optical and electrical mechanisms. Photocarrier dynamics set reservoir states, whereas ferroelectric polarization programs weights, enabling independent optimization.

**Fig. S3.** Fabrication process flow of the ferroelectric vertical organic field-effect transistor (Fe-vOFET).

**Fig. S4. a** Schematic diagram (upper panel) and optical microscopy image (lower panel) of a typical Fe-vOFET. **b** Atomic force microscopy (AFM) image of HfO_2_ films. **c** AFM image of a graphene monolayer grown on HfO_2_ substrate, with a measured thickness of 1 nm. **d** AFM image of a rubrene crystal with a thickness of 255 nm. **e** AFM image of F_16_-CuPc films. **f** UV-NIR absorption spectra of rubrene crystals and F_16_-CuPc films.

**Fig. S5. a** AFM image of hafnium zirconium oxide (HZO) films. **b** Out-of-plane phase images of HZO films acquired via piezoresponse force microscopy (PFM). **c** Local PFM phase loop (black curve) showing an unambiguous 180° phase shift, and corresponding butterfly-shaped amplitude loop (red curve).

**Fig. S6.** Schematic illustration of the mechanism underlying synaptic behavior in Fe-vOFETs.

**Fig. S7.** Comparison of planar and vertical transistor configurations. The vertical-channel transistor exhibits a significantly higher on/off ratio and stronger photoelectric feedback than the planar device, owing to the shortened transport path and enhanced gate–channel coupling. These improvements enrich the reservoir dynamics and widen the programmable conductance window for the readout, thereby benefiting both temporal encoding and linear weight programming.

**Fig. S8. a** The output curves of Fe-vOFETs at different temperatures. **b** The Arrhenius plot of Fe-vOFETs at different *V*_d_. **c** The extracted Schottky barrier *Φ*_SB_ as a function of *V*_d_.

**Fig. S9.** Wavelength-dependent EPSC and PPF behaviors of the device. **a-d**. Single-pulse EPSC responses (left), paired-pulse responses (middle), and PPF index versus inter-pulse interval (right) under optical stimulation at 340, 530, 625, and 780 nm, respectively. The device shows pronounced EPSC decay and paired-pulse facilitation at all wavelengths, with the PPF index gradually decreasing as the pulse interval increases, indicating wavelength-dependent short-term memory and fading dynamics.

**Fig. S10.** Ultralow-power optical reservoir operation. **a.** Transient photocurrent responses under optical stimulation at *V*_d_ = −25 μV, showing clear short-term synaptic behavior with an electrical energy dissipation as low as 52 aJ per event and an optical pulse energy of about 1.0 pJ per spike. **b.** Comparison of the energy consumption of this work with representative reported organic synaptic devices.

**Fig. S11.** Optoelectronic performance comparison of the vertical Fe-vOFET. **a.** Transient photo responses under different optical powers at 340 nm. **b.** Responsivity of the device at 340, 530, 625, and 780 nm. **c.** Transient photo responses under different optical powers at 340 nm.

**Fig. S12.** Transient photocurrent responses of the device under four consecutive optical pulses at 340, 530, 625, and 780 nm.

**Fig. S13.** Synergistic optoelectronic modulation of reservoir dynamics. **a.** Transient responses to a representative “1011” optical sequence under coupled wavelength and gate-voltage (*V*_g_) inputs, demonstrating diversified current amplitudes and an expanded dynamic range. **b.** Extracted relaxation time constant versus *V*_g_ across four distinct wavelengths. The reservoir’s fading-memory timescale is systematically tuned by the sub-coercive gate bias, providing an extra electrical degree of freedom to enrich the multi-timescale states without triggering ferroelectric switching.

**Fig. S14.** Emission spectra of the four fiber-coupled LEDs used in the revised optical measurements. Normalized emission spectra of the LEDs centered at 340, 530, 625, and 780 nm. The well-defined spectral outputs ensure more reliable wavelength-dependent transient photo response characterization.

**Fig. S15.** Statistical distributions of nonvolatile conductance states for the ANN/readout module. **a.** Repeated readout traces of representative programmed conductance states. **b.** Statistical distributions of the corresponding conductance states based on 500 read points per state, showing overall state separation.

**Fig. S16.** Performance comparison between devices with MFS and MFMIS structures. **a, b**. Schematics and *I*_d_-*V*_g_ sweeping characteristics (100 cycles) of the proposed MFMIS (a) and conventional MFS (b) structures. The MFMIS stack effectively prevents the severe hysteresis degradation caused by direct interfacial charge injection in MFS devices. **c, d.** Retention (c) and endurance (d) performances. Protected by the dielectric buffer layer, the MFMIS device sustains a robust memory window over extended time and >10^6^ switching cycles. **e.** Conductance modulation. The MFMIS architecture achieves a larger dynamic range with significantly improved linearity and symmetry for reliable weight programming.

**Fig. S17.** Low-bias readout characteristics. **a, b.** Potentiation and depression characteristics measured under reduced *V*_d_.

**Fig. S18.** Transfer curves of 21 devices.

**Fig. S19.** Decoupled optical and ferroelectric operation in the Fe-vOFET. **a.** Programmed conductance states under repeated optical pulse stimulation. **b.** Recovery of the original nonvolatile baseline after optical excitation. **c.** PFM phase images showing electrical switching but no observable light-induced change in ferroelectric domains.

**Fig. S20.** Relaxation dynamics of the Fe-vOFET under a single 650 nm optical pulse at different gate voltages, with multiple pulse intensities probed at each gate bias, demonstrating that the transient photocurrent decay can be modulated by gate bias.

**Fig. S21. a** Recognition accuracy for three readout strategies: experimental LTP/LTD conductance mapping, completely linear symmetric ideal synapse, and software weights without mapping. The recognition accuracy obtained using the experimental device weights (91.95% ± 2.50%) is close to that obtained using ideal linear-symmetric weights (92.72% ± 2.48%) and software weights (93.92% ± 2.03%), indicating the excellent weight-storage and update characteristics of the device. **b** Recognition accuracy versus the number of principal components. The recognition accuracy gradually increases with the number of principal components, reaching the maximum at 40, and then gradually saturates and decreases, which may be due to the redundancy of feature information. Hence, performance saturates near 40, balancing information retention and redundancy.

**Fig. S22. a.** Validation accuracy as a function of epoch under extended training. **b**. Training loss as a function of epoch.

**Fig. S23. a** Recognition accuracy with reservoir (16 RC states) versus without reservoir (binary 0/1 input). **b** Confusion matrix of the baseline configuration without reservoir.

**Fig. S24. a** MNIST preprocessing and encoding. The input binarized image is cropped and converted to input pulse trains. The image is partitioned column -wise into seven contiguous parts, each containing four pixels. Each encoded pulse train is then applied sequentially to the Fe-vOFET-based physical reservoir. Then principal component analysis (100 principal components are reserved in each sub-reservoir) and ANN are implemented as same as the process in Figure 4a. **b** Effect of grouped sub-reservoir number on recognition accuracy. Accuracy is plotted versus the number of wavelength-grouped sub-reservoirs. Increasing the number of groups expands effective dimensionality and improves classification.

**Fig. S25.** CIFAR-10 validation of the photoelectric RC framework. **a.** Representative CIFAR-10 input image, extracted feature sequence, and the corresponding encoded 4-bit pulse map. **b.** Recognition accuracy for four representative CIFAR-10 classes. **c.** Summary of the classification results for the selected categories.

**Table S1.** Comparison of reservoir computing systems based on different device platforms.

| Device platform | | Integration level | Reservoir dynamics | Readout characteristics | Demonstrated tasks | Ref. |
| --- | --- | --- | --- | --- | --- | --- |
| Memristors | Separate devices | | Single timescale | Digital (software) | Static/dynamic vision | ^12^ |
| Luminescent synaptic | Separate devices | | Single timescale | Digital (software) | dynamic vision | ^13^ |
| Memristors | Separate devices | | Multi-timescale | Digital (software) | Static/dynamic vision | ^14^ |
| v-OFET | Separate devices | | Multi-timescale | Digital (software) | Static/dynamic vision | ^15^ |
| OEST | Separate devices | | Multi-timescale | Digital (software) | Static vision | ^16^ |
| Photo-memristors | Separate devices | | Multi-timescale | Digital (software) | Static/dynamic vision | ^17^ |
| Memristors | Separate devices | | Multi-timescale | Digital (software) | Static vision | ^18^ |
| light-emitting synaptic | Separate devices | | Multi-timescale | Digital (software) | Static vision | ^19^ |
| T-FET | Separate devices | | Multi-timescale | Digital (software) | Static vision | ^20^ |
| Memristor  (WO_X_ + TiO_X_) | Separate devices | | Single timescale | Moderately nonlinear (device-conductance) | Static vision | ^21^ |
| Ph-TFET + memristors | Separate devices | | Single timescale | Moderately nonlinear (device-conductance) | Static vision | ^22^ |
| FDs (different processes) | Separate devices | | Multi-timescale | Moderately nonlinear (device-conductance) | Static vision | ^23^ |
| Ferroelectric + redox-based memristor | Separate devices | | Single timescale | Linear and symmetric  (device-conductance) | Static vision | ^24^ |
| Fe-FET with different phases | Separate devices | | Multi-timescale | Linear and symmetric  (device-conductance) | Static vision | ^25^ |
| Fe-FET | Single device | | Single timescale | Moderately nonlinear (device-conductance) | Static vision | ^26^ |
| Memristors | Single device | | Single timescale | Linear and symmetric  (device-conductance) | Static vision | ^27^ |
| OEST | Single device | | Multi-timescale | Moderately nonlinear (device-conductance) | Static vision | ^28^ |
| Fe-vOFET | Single device | | Multi-timescale | Linear and symmetric  (device-conductance) | Static/dynamic vision | This work |

Notes: v-OFET denotes a vertical organic field-effect transistor; OEST denotes an organic electrochemical synaptic transistor; T-FET denotes a thin-film field-effect transistor; and FDs denote ferroelectric diodes. In the column “Integration level”, “Separate devices” indicates that the reservoir and readout are implemented using different device structures, whereas “Single device” indicates that both functions are realized within a unified device architecture, which is favourable for system-level integration. For “Readout characteristics”, “Digital (software)” refers to cases where the readout layer is implemented purely in software without explicit device modelling. In the remaining entries, the readout weights are obtained by mapping software-optimized weights onto experimentally measured conductance-update curves of the corresponding devices. Because the linearity and symmetry of these conductance updates strongly influence the final recognition accuracy, we evaluate them using the asymmetry nonlinearity factor (ANL): systems with ANL < 0.35 are labelled “Linear and symmetric”, whereas those with ANL > 0.35 are labelled “Moderately nonlinear”. The “Demonstrated tasks” column summarizes whether each work demonstrates static and/or dynamic vision applications, where static vision refers to tasks such as handwritten-digit or fingerprint recognition and dynamic vision refers to time-dependent tasks such as human-action recognition or traffic prediction.

Note S1. Device preparation and film characterizations

The active organic channel was implemented as a planar rubrene/F_16_-CuPc heterojunction. Single-crystal rubrene was adopted for its high hole mobility and long exciton-diffusion length, which support efficient carrier transport and photogating. F_16_-CuPc provides complementary absorption from the ultraviolet to the visible region and a deep electron affinity, and the pair forms a type-II band alignment that facilitates exciton dissociation and charge separation at the interface. The heterojunction therefore delivers broadband optical response suitable for multiwavelength operation in the vertical OFET.

Fig. S4a shows a schematic of the vertical OFET and an optical micrograph of a representative device. A monolayer graphene film, prepared by mechanical exfoliation, was dry-transferred onto an HfO_2_-coated substrate. Atomic force microscopy (AFM) height profiles confirmed a thickness of approximately 1 nm (Fig. S4c). Rubrene single crystals grown by physical vapor transport were then laminated onto the graphene to define the vertical channel. A channel thickness of 255 nm was selected to balance a high on-state current with a low off-state current (Fig. S4d). Subsequently, a 10 nm F_16_-CuPc layer was thermally evaporated onto rubrene to form the planar rubrene/F_16_-CuPc heterojunction. The F_16_-CuPc morphology depended strongly on the underlying template. On rubrene single crystals it formed millimeter-scale stripe-like crystallites with a root-mean-square roughness of 2.12 nm (Fig. S4e), whereas on SiO_2_ without the rubrene template it formed random granular films with roughness typically above 5 nm. Finally, gold top electrodes were transferred onto the F_16_-CuPc layer to complete the device.

AFM measurements showed a very smooth HZO surface with a root-mean-square roughness of 0.56 nm (Fig. S5a). Piezo response force microscopy (PFM) confirmed ferroelectric behavior. Domain-writing at ±10 V produced amplitude and phase maps with an approximately 180° phase contrast between written regions, indicating reversible remanent polarization (Fig. S5b). Local PFM spectroscopy under ±10 V produced a complete set of phase-hysteresis and amplitude-butterfly loops, further verifying robust ferroelectric switching in the HZO film (Fig. S5c).

Note S2. Extraction of the Schottky barrier at the contacts

Temperature-dependent current–temperature measurements were performed at a set of fixed biases to extract the injection barrier at the graphene/rubrene contact. In the thermionic-emission regime the current follows:

$\boldsymbol{I}_{\mathbf{ds}}\boldsymbol{=A}\boldsymbol{A}^{\boldsymbol{*}}\boldsymbol{T}^{\boldsymbol{2}}\mathbf{exp}\boldsymbol{(-q}\boldsymbol{\Phi}_{\mathbf{SB}}\boldsymbol{/}\boldsymbol{k}_{\mathbf{B}}\boldsymbol{T)}$	(1)

where *A* is the effective area, *A*^*^ is the Richardson constant, *q* is the elementary charge, *k*_B_ is Boltzmann’s constant, and *Φ*_SB_ is the Schottky barrier height at bias *V*. Plotting

ln(I/*T*²) versus 1/*T* yields straight lines whose slopes are −*qΦ*_SB_(*V*)/*k*_B_; linear fits to these Arrhenius plots for each bias give *Φ*_SB_(*V*). As temperature decreases the current drops markedly, consistent with thermally activated transport. The extracted *Φ*_SB_(*V*) decreases approximately linearly with increasing voltage, in line with field-induced barrier lowering, and extrapolation to zero bias gives a zero-bias barrier *Φ*_SB, 0_ ≈137.2 meV for the graphene/rubrene interface. The high hole-injection barrier at graphene/rubrene accounts for the lower off-state current. When a positive gate bias is applied and graphene acts as the drain, hole depletion near the interface increases band bending and suppresses injection. Under negative gate bias, hole accumulation reduces band bending and the drain current rises. These trends indicate that the gate modulates the Schottky barrier and the associated thermionic-emission current, consistent with the barrier values extracted from the Arrhenius analysis.

Note S3. Evaluation of linearity and symmetry of conductance update behaviours

Weight updates were driven by a fixed-amplitude biphasic pulse protocol as defined in the main text. For potentiation we applied +3 V pulses of 200 μs, and for depression we applied −5 V pulses of 200 μs. We then quantified the linearity of LTP and LTD using the following expressions in order to assess whether the readout layer meets the required linearity.

$G_{\mathrm{pot}}=B\left( 1-e^{\frac{-P}{A_{\mathrm{pot}}}} \right)+G_{\min}$	(1)

$G_{\mathrm{dep}}=-B\left( 1-e^{\frac{P-P_{\max}}{A_{\mathrm{dep}}}} \right)+G_{\max}$	(2)

$B=(G_{\max}-G_{\min})/\left( 1-e^{\frac{-P_{\max}}{A_{dep, dep}}} \right)$	(3)

In these expressions, *G*_pot_ and *G*_dep_ denote the conductance during potentiation and depression. *P*_max_ is the total pulse count and *P* the pulse index. *G*_min_ and *G*_max_ set the conductance bounds. *A*_pot_ and *A*_dep_ quantify the update linearity for potentiation and depression, with *A*=1 indicating an ideal linear trajectory. Values below 1 are concave and values above 1 are convex. Fits were obtained by nonlinear least squares with monotonicity enforced. Linearity, defined as how closely the conductance as a function of pulse count follows a straight-line trajectory, was assessed for MFS and MFMIS gate stacks under the same fixed-pulse protocol. Replacing MFS with MFMIS improved the indices from *A*_pot_ = 0.36 ± 0.07 and *A*_dep_ =1.61 ± 0.12 to *A*_pot_ = 0.80 ± 0.04, *A*_dep_ = 1.15 ± 0.08 (Fig. S16e). The asymmetry non-linearity factor, calculated as the equation:

$ANL=(G_{\mathrm{pot}}(P_{\max}/2)-G_{\mathrm{dep}}(P_{\max}/2))/\left( G_{\max}-G_{\min} \right)$	(4)

which equals 0.26 ± 0.05 for the MFMIS configuration. A smaller value indicates updates that are closer to linear and symmetric. The MFMIS gate stack introduces an intermediate metal and a gate insulator. This configuration improves electrostatic coupling and charge compensation during polarization switching, reduces direct interaction between the ferroelectric and the semiconductor channel, and limits leakage and read-disturb. These effects mitigate depolarization and interfacial trapping, which supports more uniform domain switching per pulse and yields trajectories that are closer to linear and symmetric under the same bias conditions.

Note S4. Power-consumption analysis of the Fe-vOFET under low-*V*_d_ operation in reservoir and readout modes

To further evaluate the low-power operating potential of the Fe-vOFET, we examined the device behaviour under reduced drain bias in both the optical reservoir mode and the electrical readout mode.

For the optical reservoir mode, the device still exhibits clear light-triggered transient responses even at an ultralow drain bias of *V*_d_ = −25𝜇V (**Fig. S10a**). Under this condition, the electrical energy dissipation of a single event can be estimated by *E* = *V*_d_ × *I*× *t*, where 𝐼 is the representative transient current and *t* is the pulse duration. Based on the measured response under the present test condition, the electrical energy dissipation is as low as 52 aJ per event. In addition, the optical excitation energy for generating an individual stimulation pulse is approximately 1.0 pJ per spike. We further compared the energy consumption of the present device with representative recently reported organic synaptic devices (**Fig. S10b**), which shows that the Fe-vOFET remains highly competitive in the low-power regime. These results indicate that the device can maintain reservoir-relevant transient dynamics even under extremely low drain bias, supporting its potential for ultralow-power optical reservoir computing and edge-intelligent sensing.

For the electrical readout mode, we further measured the conductance-update characteristics at *V*_d_ = −50 mV (**Fig. S17**). The device still preserves the essential LTP/LTD-type conductance modulation required for the readout layer, confirming that the ferroelectric programming function remains operable under reduced drain bias. At the same time, lowering *V*_d_ compresses the effective conductance window, which leads to a reduced on/off ratio, less ideal update linearity, and smaller separation between adjacent conductance states. The symmetry of potentiation and depression may also be slightly affected. Physically, this behaviour can be understood from the weaker drain-side current modulation at lower *V*_d_, which reduces the dynamic range available for conductance update. Therefore, reduced drain bias is beneficial for lowering electrical energy consumption, but excessively small *V*_d_ also makes accurate weight representation more challenging.

Taken together, these results show that the Fe-vOFET supports low-power operation in both functional modes, although the optimization criteria are different. In the reservoir mode, the key requirement is to preserve sufficiently distinguishable transient optical responses under ultralow bias. In the readout mode, the main consideration is to balance reduced power consumption with adequate conductance dynamic range, linearity, and state separability for reliable weight storage and update.

Note S5. Scalability and system-level utilization of the Fe-vOFET for neuromorphic vision hardware

Although the present work focuses on a single-device proof-of-concept, the proposed Fe-vOFET is intended as a device platform that can be extended toward more practical neuromorphic vision systems. A natural system-level architecture is to construct both a reservoir array and a readout array using the same Fe-vOFET platform operated in different modes. In such a scheme, the reservoir array would provide high-dimensional temporal features through nonlinear volatile optical dynamics, whereas the ferroelectric readout array would provide multiple output nodes through non-volatile weight storage and parallel weighted summation.

At the array level, standard row/column addressing and parallel current readout could in principle be adopted, similar to other transistor-array-based neuromorphic systems. In this framework, the number of effective output weights scales with the size of the readout array, while higher-dimensional feature extraction can be achieved by increasing the number of reservoir nodes, temporal sampling points, wavelength channels, or spatial input channels. Therefore, the present single-device operation can be viewed as the elementary functional unit of a larger reservoir–readout hardware system. Several practical considerations arise when extending the architecture to larger scale. First, fan-out and interconnect routing become increasingly important as the number of reservoir outputs and readout channels grows. Efficient array layout and signal routing would be required to preserve the compactness advantage of the monolithic platform. Second, device-to-device variability may affect the consistency of both transient reservoir dynamics and programmed readout states, making calibration and array-level compensation strategies important for robust large-scale implementation. Third, the programming overhead of the ferroelectric readout array must also be considered, especially when many output weights need to be updated during training or reconfiguration. In this regard, the non-volatile character of the ferroelectric readout is advantageous because the programmed weights can be retained without continuous refreshing.

A key advantage of the present Fe-vOFET platform is that both reservoir and readout functions are implemented using the same device technology, rather than relying on heterogeneous integration of different device types. This feature can simplify fabrication flow, reduce interface incompatibility, and facilitate compact integration of the sensing, reservoir, and readout modules. Therefore, while further array-level engineering will be required to address practical issues such as variability, interconnect, and write overhead, the Fe-vOFET provides a promising hardware basis for scalable neuromorphic vision modules with mode-separated reservoir and readout operation.

Note S6. Quantitative comparison of the vertical Fe-vOFET with representative planar and vertical organic synaptic transistors

To better contextualize the role of the vertical channel architecture, we compared the Fe-vOFET with representative reported planar and vertical organic synaptic transistors in terms of key optoelectronic metrics, including responsivity, response timescale, and energy consumption. The Fe-vOFET exhibits responsivities of 117.2 ± 12.1 A/W, 66.8 ± 8.3 A/W, 37.6 ± 6.4 A/W, and 13.4 ± 3.7 A/W at 340, 530, 625, and 780 nm, respectively (**Fig. S11a**, **b**). These values place the device in a competitive range relative to representative planar and other vertical organic synaptic transistors, while also highlighting its broad spectral synaptic photo response from the UV to the near-infrared range. The advantage of the vertical architecture is not reflected only in responsivity, but also in the balance among multiple performance metrics. The short carrier-transport path in the vertical channel is favourable for efficient current modulation and low-bias operation, while the compact geometry supports strong optical response and a high on/off ratio. In addition, as discussed in the main text and Note S4, the device can still maintain clear optical reservoir responses at *V*_d_ = −25 μV, corresponding to an electrical energy dissipation as low as 52 aJ per event, with an optical stimulation energy of approximately 1.0 pJ per spike.

At the same time, the response time of the device is in the millisecond regime, which is consistent with the temporal processing tasks targeted in this work. In principle, the short carrier-transport path in the vertical channel is suitable for faster operation and could support shorter optical pulse intervals. However, when the pulse interval is reduced, the photocurrent amplitude also decreases, which in turn weakens the contrast and richness of the reservoir states (**Fig. S11c**). Therefore, although the vertical geometry provides the basis for faster transport, the effective operating timescale of the optical reservoir is ultimately determined by a balance between transport efficiency and the need to preserve sufficiently distinguishable transient states for reservoir computing.

**References**

1.	Wu, X. et al. Ultralow-power optoelectronic synaptic transistors based on polyzwitterion dielectrics for in-sensor reservoir computing. Sci. Adv. **10**, eadn4524 (2024).

2.	Chen, T. et al. Photonic synapses with ultra‐low energy consumption based on vertical organic field‐effect transistors. Adv. Optical Mater. **9**, 2002030 (2021).

3.	Zhang, Q. et al. Ultralow‐power vertical transistors for multilevel decoding modes." Adv. Mater. **35**, 2208600 (2023).

4.	Liu, G. et al. Ultralow‐power and multisensory artificial synapse based on electrolyte‐gated vertical organic transistors. Adv. Funct. Mater. **32**, 2200959 (2022).

5.	Wu, J. et al. Low‐Power and Multimodal Organic Photoelectric Synaptic Transistors Modulated by Photoisomerization for UV Damage Perception and Artificial Visual Recognition. Adv. Funct. Mater. **35**, 2420073 (2025).

6.	Li, D. et al. Insulating Electrets Converted from Organic Semiconductor for High‐Performance Transistors, Memories, and Artificial Synapses. Adv. Funct. Mater. **33**, 2305012 (2023).

7.	Shi, J. et al. A fully solution‐printed photo synaptic transistor array with ultralow energy consumption for artificial‐vision neural networks. Adv. Mater. **34**, 2200380 (2022).

8.	Li, L. et al. Autonomous light intensity adaptation in an energy‐efficient retinomorphic organic ferroelectric neuristor. Adv. Optical Mater. **12**, 2303172 (2024).

9.	Chen, Y. et al. Autonomous Decision-Making Machine Vision System Enabled by a Low-Voltage, Photo adaptive Organic Synaptic Transistor. Nano Lett. (2026).

10.	Wu, X. et al. Highly sensitive, low-energy-consumption biomimetic olfactory synaptic transistors based on the aggregation of the semiconductor films. ACS Sens. **9**, 2673-2683 (2024).

11. Liu, C. et al. Bipolar synaptic organic/inorganic heterojunction transistor with complementary light modulation and low power consumption for energy-efficient artificial vision systems. Sci. China Mater. **67**, 1500-1508 (2024).

12.	Sun, Y. et al. In‐Sensor Reservoir Computing Based on Optoelectronic Synapse. Adv. Intell. Syst. **5**, 2200196 (2023).

13.	Wi, S. et al. Multi-Color Synaptic Luminescence in RE-Doped Ca2SnO4 (RE = Sm3+, Er3+, and La3+). Adv. Funct. Mater. **35**, 2414860 (2025).

14.	Jang, Y. H. et al. A high-dimensional in-sensor reservoir computing system with optoelectronic memristors for high-performance neuromorphic machine vision. Mater. Horiz. **11**, 499–509 (2023).

15.	Gao, C. et al. Toward grouped-reservoir computing: organic neuromorphic vertical transistor with distributed reservoir states for efficient recognition and prediction. Nat. Commun. **15**, 740 (2024).

16.	Lee, K. H. et al. Temporally Reconfigurable Reservoir Computing with Flexible Electrolyte-Gated TFTs for High-Performance Neuromorphic Processing. Adv. Mater. e07979 (2025).

17.	Tan, H. & van Dijken, S. Dynamic machine vision with retinomorphic photomemristor-reservoir computing. Nat. Commun. **14**, 2169 (2023).

18.	Yamazaki, Y. & Kinoshita, K. Photonic Physical Reservoir Computing with Tunable Relaxation Time Constant. Adv. Sci. **11**, 2304804 (2024).

19.	Lian, M. et al. Towards mixed physical node reservoir computing: light-emitting synaptic reservoir system with dual photoelectric output. Light Sci. Appl. 13, 179 (2024).

20.	Chen, R. et al. Thin-Film Transistor for Temporal Self-Adaptive Reservoir Computing with Closed-Loop Architecture. Sci. Adv. **10**, eadl1299 (2024).

21.	Singh, A., Choi, S., Wang, G., Daimari, M. & Lee, B. G. Analysis and fully memristor-based reservoir computing for temporal data classification. Neural Networks **182**, 106925 (2025).

22.	Zhang, Z. et al. In-sensor reservoir computing system for latent fingerprint recognition with deep ultraviolet photo-synapses and memristor array. Nat. Commun. **13**, 6590 (2022).

23.	Chen, Z. et al. All-ferroelectric implementation of reservoir computing. Nat. Commun. **14**, 3585 (2023).

24.	Lee, J. K. et al. Integrated Design of Electrically Configurable Ferroelectric and Redox-Based Memristors for Hardware-Implemented Reservoir Computing. Adv. Sci. **12**, e05688 (2025).

25.	Kim, J. et al. Analog reservoir computing via ferroelectric mixed phase boundary transistors. Nat. Commun. **15**, 9147 (2024).

26.	Li, J. et al. Phase-Engineered In2Se3 Ferroelectric P-N Junctions in Phototransistors for Ultra-Low Power and Multiscale Reservoir Computing. ACS Nano **19**, 13220–13229 (2025).

27.	Zhou, J. et al. Optoelectronic polymer memristors with dynamic control for power-efficient in-sensor edge computing. Light Sci. Appl. **14**, 309 (2025).

28.	Liu, R. et al. Hardware-Feasible and Efficient N-Type Organic Neuromorphic Signal Recognition via Reservoir Computing. Adv. Mater. 37, 2409258 (2025).
